# *Lactiplantibacillus plantarum*-Derived Biosurfactant Attenuates Quorum Sensing-Mediated Virulence and Biofilm Formation in *Pseudomonas aeruginosa* and *Chromobacterium violaceum*

**DOI:** 10.3390/microorganisms10051026

**Published:** 2022-05-13

**Authors:** Mitesh Patel, Arif Jamal Siddiqui, Syed Amir Ashraf, Malvi Surti, Amir Mahgoub Awadelkareem, Mejdi Snoussi, Walid Sabri Hamadou, Fevzi Bardakci, Arshad Jamal, Sadaf Jahan, Manojkumar Sachidanandan, Mohd Adnan

**Affiliations:** 1 Department of Biotechnology, Parul Institute of Applied Sciences and Centre of Research for Development, Parul University, Vadodara 391760, India; patelmeet15@gmail.com; 2 Department of Biology, College of Science, University of Hail, Hail P.O. Box 2440, Saudi Arabia; arifjamal13@gmail.com (A.J.S.); snmejdi@yahoo.fr (M.S.); walidsabrimail@gmail.com (W.S.H.); fevzi.bardakci@gmail.com (F.B.); arshadjamalus@yahoo.com (A.J.); 3 Department of Clinical Nutrition, College of Applied Medial Sciences, University of Hail, Hail P.O. Box 2440, Saudi Arabia; amirashrafy2007@gmail.com (S.A.A.); mahgoubamir22@gmail.com (A.M.A.); 4 Bapalal Vaidya Botanical Research Centre, Department of Biosciences, Veer Narmad South Gujarat University, Surat 395007, India; malvisurti92@gmail.com; 5 Department of Medical Laboratory Sciences, College of Applied Medical Sciences, Majmaah University, Al-Majmaah 11952, Saudi Arabia; s.jahan@mu.edu.sa; 6 Department of Oral Radiology, College of Dentistry, University of Hail, Hail P.O. Box 2440, Saudi Arabia; smanojk68@gmail.com

**Keywords:** quorum sensing, antibiofilm, biosurfactant, violacein, pyocyanin, acyl homoserine lactone, GC–MS, glycolipid, exopolysaccharide

## Abstract

Quorum sensing (QS) controls the expression of diverse biological traits in bacteria, including virulence factors. Any natural bioactive compound that disables the QS system is being considered as a potential strategy to prevent bacterial infection. Various biological activities of biosurfactants have been observed, including anti-QS effects. In the present study, we investigated the effectiveness of a biosurfactant derived from *Lactiplantibacillus plantarum* on QS-regulated virulence factors and biofilm formation in *Pseudomonas aeruginosa* and *Chromobacterium violaceum*. The structural analogues of the crude biosurfactant were identified using gas chromatography–mass spectrometry (GC–MS). Moreover, the inhibitory prospects of identified structural analogues were assessed with QS-associated CviR, LasA, and LasI ligands via in silico molecular docking analysis. An *L. plantarum*-derived biosurfactant showed a promising dose-dependent interference with the production of both violacein and acyl homoserine lactone (AHL) in *C. violaceum*. In *P. aeruginosa*, at a sub-MIC concentration (2.5 mg/mL), QS inhibitory activity was also demonstrated by reduction in pyocyanin (66.63%), total protease (60.95%), LasA (56.62%), and LasB elastase (51.33%) activity. The swarming motility and exopolysaccharide production were also significantly reduced in both *C. violaceum* (61.13%) and *P. aeruginosa* (53.11%). When compared with control, biofilm formation was also considerably reduced in *C. violaceum* (68.12%) and *P. aeruginosa* (59.80%). A GC–MS analysis confirmed that the crude biosurfactant derived from *L. plantarum* was a glycolipid type. Among all, n-hexadecanoic acid, oleic acid, and 1H-indene,1-hexadecyl-2,3-dihydro had a high affinity for CviR, LasI, and LasA, respectively. Thus, our findings suggest that the crude biosurfactant of *L. plantarum* can be used as a new anti-QS/antibiofilm agent against biofilm-associated pathogenesis, which warrants further investigation to uncover its therapeutic efficacy.

## 1. Introduction

The spread of antibiotic resistance nowadays is more rapid than ever before. It is a serious concern in society that bacteria are becoming resistant to antibacterial compounds used to treat clinical infections [1]. In order to develop such resistance, some bacteria form biofilms [2]. Microorganisms attach themselves to living or nonliving surfaces via biofilms, allowing them to remain resistant after producing polymeric substances. Inside biofilms, microbial cells are protected from cellular responses, antimicrobial treatments, and adverse environmental conditions [3]. Furthermore, bacteria-producing biofilms are much more resistant to antibiotics, as the biofilm matrix prevents antibiotics from penetrating deeply into the bacterial cells. In order to control the attack of pathogenic bacteria that form biofilms, it is necessary to identify microorganisms and compounds that are capable of inhibiting or destroying the biofilm [4].

Quorum sensing (QS) describes the process of detecting the number of microorganisms in a population and controlling the gene expression after the number reaches a certain threshold [5]. The QS mechanism is used by some pathogenic bacteria to develop biofilms. QS involves the exchange of signals between bacteria through extracellular molecules called autoinducers. As a result of these signal molecules, bacteria are able to express virulence factors, produce secondary metabolites, form biofilms, and communicate with other microorganisms [6]. As part of the QS process, signal molecules from bacteria bind to other bacteria’s receptors, and genes that are transcribed will enable communication within and between species [7]. Furthermore, virulence factors also contribute to the regulation of cellular functions, such as resistance to antibiotics, formation of spores, production of toxins, and regulation of mobility [5,8]. Bacterial infections can be controlled by QS inhibition (QSI), since it prevents the growth of bacterial biofilms and reduces their virulence [9]. A number of recent studies have demonstrated that the QS mechanism and the development of bacterial resistance are intimately linked [10]. Thus, inhibiting the QS mechanism represents a potentially promising new antibacterial strategy that can not only inhibit bacterial resistance development but also inhibit the expression of virulence genes associated with population density within a given pathogen population [5].

A group of bacteria called lactic acid bacteria (LAB) are commonly known as “probiotic bacteria”. They play an essential role in the health and immunity of human beings. Diverse vital metabolites are produced by LAB, including hydrogen peroxide, acetic acid, lactic acid, bacteriocin, and biosurfactants, which provide protection and have antagonistic effects [11,12]. The ability of a few LAB to produce biosurfactants and their various applications, such as in the inhibition of microbial adhesion, desorption activity, and biofilm inhibition on a variety of surfaces, such as polypropylene, rubber, silicone, and different medical devices/instruments and implants, has been reported in a number of studies [13,14,15,16,17,18,19,20,21]. Recently, we reported that a few LAB strains can efficiently produce biosurfactants, and demonstrated the antibacterial and antibiofilm potential of a biosurfactant produced from LAB [22,23]. In this study, we hypothesized that LAB may also be a prospective source of novel anti-QS compounds. Therefore, we studied the anti-QS and antibiofilm potential of a crude biosurfactant derived from *Lactiplantibacillus plantarum* (*L. plantarum*) against *Chromobacterium violaceum* (*C. violaceum*) (MTCC2656) and *Pseudomonas aeruginosa* (*P. aeruginosa*) (MTCC2488) at a broad-spectrum level.

## 2. Materials and Methods

### 2.1. Isolation and Identification of Lactic Acid Bacteria

Isolation of probiotic LAB was made using a yoghurt sample, and preparation was performed by transferring the yoghurt sample into a conical flask containing 100 mL of de Man, Rogosa, and Sharpe (MRS) broth (HiMedia^®^, Mumbai, India), and the selected MRS media were used as enrichment media. After incubation, an enriched sample (100 μL) was spread over the MRS agar plates, and incubation was performed at 37 °C for 48 h under anaerobic conditions. Afterwards, purified colonies of bacteria were sub-cultured for further use. For immediate use, the purified colonies of bacteria kept on MRS agar media, and subsequently for later use, were kept in 20% glycerol at −20 °C. A 16S rRNA gene sequencing method was applied for the identification of isolated LAB. Furthermore, the sequence match analysis of the identified LAB was carried out using the basic local alignment search tool (BLAST) on NCBI, and the obtained sequence was submitted to GenBank [22].

### 2.2. Production and Extraction of Biosurfactant

Isolated and purified LAB stains were used for the production of a biosurfactant using an MRS-Lac broth (specific LAB culture medium, where lactose is used as sugar instead of glucose). Furthermore, the crude biosurfactant was developed using an *L. plantarum* (1%) culture grown overnight at 37 °C for 48 h without shaking by means of the MRS-Lac (1000 mL) medium. To harvest the cells, the culture medium was centrifuged (10,000 rpm, 10 min, 10 °C) after the incubation period ended. After washing the cells in twofold times with demineralized water, they were again suspended in 100 mL of phosphate-buffered saline (PBS) (pH 7.0). To release the biosurfactant associated with the cells, the solution was agitated gently at room temperature for 4 h. Afterwards, the prepared samples were centrifuged to remove the bacterial cell, and collection of the supernatant was performed by filtering the sample solution with 0.22 µm filter paper. Lastly, the collected supernatant was lyophilized and stored at −40 °C before being resuspended in deionized water at 100 mg/mL, and the obtained crude biosurfactant solution was further used for biological assays [23].

### 2.3. Biosurfactant Assays

The confirmation of a cell-bound biosurfactant produced by isolated LAB was carried out using various quantitative and qualitative assays, such as emulsification assay, drop collapse assay, oil spreading assay, and blue agar plate (BAP) assay per our previous studies [22,23].

#### 2.3.1. Bacterial Strains and Growth Conditions

The bacterial strains used in this study are *C. violaceum* (MTCC2656) and *P. aeruginosa* (MTCC2488) to check the QS inhibitory potential of a crude biosurfactant of *L. plantarum*. The selected bacterial strains were cultured using a Luria-Bertani (LB) broth (HiMedia^®^, Mumbai, India) at 30 °C for 24 h with shaking at 120 rpm.

#### 2.3.2. Determination of Minimum Inhibitory Concentration (MIC)

A crude biosurfactant developed from *L. plantarum* and its MIC were determined by using a broth dilution method [24]. The active culture of bacteria in an LB broth was used to prepare inocula. In a 96-well plate (100 µL each well), the obtained crude biosurfactant was diluted twofold ranging from 100 to 1.56 mg/mL in LB.

Furthermore, the diluted culture of each test bacterium was added to the respective wells to make up a final concentration of 10^8^ CFU/mL, followed by incubation for 24 h at 37 °C. The MIC value was then noted down as at what concentration growth was inhibited. A negative control consisted of only media, while a positive control consisted of only inoculated bacteria but no biosurfactant.

### 2.4. Quorum Sensing Inhibitory Activity in C. violaceum

#### 2.4.1. Evaluation of Anti–Quorum Sensing Activity

An evaluation of the anti-QS activity of the crude biosurfactants of *L. plantarum* was carried out via well diffusion assay using *C. violaceum*. A bacterial culture (100 µL) of *C. violaceum* was grown overnight and spread over the LB plates, and wells were made with the help of a cork borer. Then, 60 µL of crude biosurfactants (100 mg/mL) was transferred and inoculated into each well, and plates were incubated at 37 °C for 24 h. After 24 h, the inhibition zone was determined, which showed anti-QS effect on tested bacteria [25].

#### 2.4.2. Violacein Inhibition Assay

In the presence and lack of a crude biosurfactant of *L. plantarum*, a violacein pigment produced by *C. violaceum* was extracted and quantified spectrophotometrically [26]. A bacterial culture grown for 16–18 h (OD_600_ nm = 0.1) was incubated in conical flasks filled with an LB broth either in the absence or in the presence of a crude *L. plantarum* biosurfactant (sub-MIC-0.5, 1 and 2 mg/mL) and incubated at 28 °C for 24 h. After incubation, a pellet of bacterial cells was collected, dissolved in 1 mL DMSO, and used for quantification. A pellet of bacterial cell solution was centrifuged at 10,000 rpm for 10 min, leading to the removal of cell debris, and the soluble violacein absorbance was measured at 585 nm. The percentage of the treated *C. violaceum* was compared with that of the control sample, while taking absorbance at 600 nm. The amount of inhibition of violacein production in the presence of the crude *L. plantarum* biosurfactant was determined as:

% violacein inhibition = (OD_600_ of control − OD_600_ of treated/OD_600_ of control) × 100.

#### 2.4.3. Quantification of Acyl Homoserine Lactones (AHLs)

The estimation of AHLs was performed per the method described by Taghadosi et al. (2015) [27] using culture supernatants of *C. violaceum* treated with or without the crude biosurfactant of *L. plantarum*. Furthermore, 20 µL of the *C. violaceum* culture was mixed in (with (sub-MIC-0.5, 1 and 2 mg/mL) or without the crude biosurfactant of *L. plantarum*) along with 180 µL of LB medium and incubated for 24 h at 30 °C under shaking condition. Afterwards, cell supernatants were collected into separate new tubes, and an identical volume of acidified ethyl acetate (0.5% glacial acetic acid) was incorporated and mixed. Then, the organic layers from control and treatment tubes were collected, concentrated, and dried using a rotary evaporator at 45 °C. Subsequently, the concentrated and dried samples were resuspended in acidified ethyl acetate (20%) and kept at −20 °C for further use. Control and treated residues were then loaded into the wells and mixed with an equal volume of hydroxyl amine (2 M)/NaOH (3.5 M). Then, equal volumes of ferric chloride (4 M HCl (10%)) and 95% ethanol mixed together were added, and absorbance was taken at 520 nm.

### 2.5. Quorum Sensing Inhibitory Activity in P. aeruginosa

#### 2.5.1. Quantitative Analysis of Pyocyanin Production in *P. aeruginosa*

The extraction of pyocyanin was performed as described by Essar et al. (1990) [28] using culture supernatants of *P. aeruginosa* treated with or without the crude biosurfactant of *L. plantarum*. Briefly, the supernatant of *P. aeruginosa* (5 mL) treated with (sub-MIC-1.5, 3.5 and 4.5 mg/mL) or without crude biosurfactants was first extracted with chloroform (3 mL), and then re-extracted with 0.2 M HCl (1 mL). Following this, the solution was transferred to a glass cuvette for the quantification of absorbance at 520 nm.

#### 2.5.2. LasA Staphylolytic Assay

In order to determine the LasA protease activity, boiled *S. aureus* cells were lysed with *P. aeruginosa* culture supernatants [29]. An *S. aureus* bacterial cell culture (10^6^ CFU/mL) grown overnight was centrifuged at 8000 rpm for 5 min, and the pellets were placed in 0.02 M Tris-HC1 (pH 8.5) solution, boiled for 10 min, and diluted with 0.02 M Tris-HCl buffer to make an optical density of 0.8 at 595 nm. Then, cell-free culture supernatants of *P. aeruginosa*, cultivated with (sub-MIC-1.5, 3.5 and 4.5 mg/mL) or without the crude biosurfactant of *L. plantarum*, were added with diluted *S. aureus* suspension (9:1 ratio). Following this, the solution was kept in a cuvette for the quantification of absorbance at 595 nm.

#### 2.5.3. LasB Elastase Assay

A method described by Adonizio et al. (2008) [30] was carried out for the measurement of elastolytic activity. According to the method, a grown *P. aeruginosa* culture was treated with crude biosurfactants of *L. plantarum* (sub-MIC-1.5, 3.5 and 4.5 mg/mL). Later on, the entire treated or control culture supernatant was transferred to 900 µL of elastin Congo red buffer (100 mM Tris, 1 mM CaCl_2_, pH 7.5) comprising 20 mg of elastin Congo red (Sigma^®^, Bengaluru, India). After 3 h of incubation at 37 °C in a shaker incubator, the insoluble components (elastin Congo red) were removed by the centrifugation process. An absorption measurement was performed by taking supernatant absorbance reading at 495 nm, and an LB medium with or without the crude biosurfactant of *L. plantarum* served as a negative control.

#### 2.5.4. Azocasein Assay for Proteolytic Activity

Using the method described by Kessler et al. (1993) [29], the proteolytic activity of supernatants without the cells of *P. aeruginosa* cultured with (sub-MIC-1.5, 3.5 and 4.5 mg/mL) or without the crude biosurfactant of *L. plantarum* was determined. After adding 150 µL of culture supernatants to 1 mL of 0.3% azocasein (Sigma^®^, St. Louis, MO, USA) in 0.05 M Tris-HCl and 0.5 mM CaCl_2_ (pH 7.5), the supernatants were incubated for 15 min at 37 °C. A 10% trichloroacetic acid (0.5 mL) was added to halt the reaction. The absorbance of the prepared sample was measured at 400 nm after centrifugation.

### 2.6. Swarming Motility Assay

*C. violaceum* and *P. aeruginosa* were tested for swarming motility using polystyrene plates containing a swarming motility medium (HiMedia^®^, India) supplemented with glucose (HiMedia^®^, India). The culture (2 µL) of tested strains grown overnight either treated (sub-MIC concentration) or untreated with the crude biosurfactant of *L. plantarum* was point-inoculated in the midpoint of plates. Afterwards, midpoint inoculated spots were allowed to dry for 20 min at room temperature and later incubated for 48 h at 37 °C. From the point of inoculation, a circle of circular bacterial growth was measured [31].

### 2.7. Extraction and Estimation of Exopolysaccharides (EPS)

The bacterial strains of *P. aeruginosa* and *C. violaceum* were grown along with the crude biosurfactant of *L. plantarum*, centrifuged for supernatants, and subsequently filter-sterilized. The supernatants were mixed with chilled absolute ethanol and kept overnight for EPS precipitate production at 4 °C [32].

### 2.8. Antibiofilm Assay

The antibiofilm potential of the crude *L. plantarum* biosurfactants at sub-MICs was determined by means of the crystal violet staining method [33]. In 96-well microtiter plates, bacterial cell cultures in LB supplemented with 0.2% of glucose (100 µL) of each test strain (10^8^ CFU/mL) and crude *L. plantarum* biosurfactant were incubated for 1 day at 37 °C. Followed by incubation time, the careful removal of planktonic cells was performed, and they were washed with PBS (200 µL). Following washing, adhered cells were stained with 0.1% crystal violet and incubated at 37 °C for 30 min to see the developed biofilms. The surplus dye was washed off with PBS, and plates were fixed with 95% ethanol (200 µL) and quantified spectrophotometrically at 470 nm.

### 2.9. In Situ Visualization of Biofilms

Biofilm visualization was performed per the method described by Musthafa et al. (2010) [34] with slight modifications to investigate the biofilms formed by test strains on glass coverslips. Microtiter plates with 24 wells consisting 1 × 1 cm coverslips were inoculated with test cultures (500 µL) in LB supplemented with 0.2% of glucose (10^8^ CFU/mL). As a treatment, 500 µL of the crude *L. plantarum* biosurfactants (sub-MIC) was added to the same well. Glass coverslips containing biofilms formed after 24 h of incubation at 37 °C were gently detached and washed with PBS. The staining of the biofilm was achieved with 0.1% crystal violet and examined under LM with a magnification of 40× (Axioscope A1, Zeiss, Jena, Germany).

### 2.10. Gas Chromatography–Mass Spectrometry (GC–MS) Analysis

GC–MS analysis and characterization were carried out as previously described [22,23]. Identification of a crude biosurfactant structural analog was achieved by using a Shimadzu Nexis GC-2030 apparatus equipped with QP2020 NX-MS. Helium (≥99.99%) as a carrier gas was applied with a flow rate of 1 mL/min. A total of 10 µL of the sample was injected into the system. The oven temperature was kept within the range of 60–260 °C. The mass spectra (MS) of the detected biosurfactant were compared with standards per the National Institute of Standards and Technology (NIST) database.

### 2.11. Molecular Docking Analysis

The structural analog of the crude biosurfactants identified by GC–MS analysis was positioned for molecular docking analysis against the QS-associated proteins of the tested strains. The crystal structures of the QS proteins, such as CviR of *C. violaceum* (PDB: 3QP5), AHL synthase LasI of *P. aeruginosa* (PDB: 1RO5), and LasA (PDB: 3IT7), were downloaded from the Protein Data Bank (RCSBPDB). The 3-D molecular structures of all the selected compounds were obtained from the PubChem database and changed into PDB format via Open Babel Software [35]. Chlorolactone was used as a standard QS inhibitor against the QS-associated CviR, LasA, and LasI ligands. All the selected compounds were docked separately against the specific receptors using AutoDock Vina. By removing the cocrystallized ligand, water molecules, and cofactors from the protein to be analyzed, the protein-linked residues were left in the file being prepared by using autopreparation of the target protein via MGL Tools 1.5.7. After the protein and ligand preparation, the graphical user interface software was used to fix the docking simulation grid box. The grid size was set to 81.663 × 118.267 × 83.264 xyz points for 3QP5, 50.322 × 45.831 × 46.224 xyz points for 1RO5, and 51.916 × 54.818 × 79.096 xyz points for 3IT7. The grid center for 3QP5 was designated at dimensions (x, y, and z): 31.056, 1.874, and 17.227; for 1RO5 (x, y, and z): 41.142, −10.485, and −13.615; and for 3IT7 (x, y, and z): 27.830, −1.027, and 8.194. The grid box was centered so as to completely enclose the binding sites of both receptors to give enough space for ligand translation and rotation. Followed by protein ligand translation rotation, as many as nine poses were considered for each ligand during the docking process. After the docking process, the analysis was made between ligands and receptors using the Discovery Studio Visualizer, and the conformations with the least free-binding energy were selected.

### 2.12. Statistical Analysis

All experiments were carried out in triplicate. Results are presented as the mean ± standard deviation (SD) of the number of experiments performed. The analysis was conducted using the software GraphPad Prism 5.0.

## 3. Results

### 3.1. Identification and Screening of Biosurfactant Production by Isolated LAB

From the yoghurt sample, a probiotic LAB strain was identified after isolation on MRS agar plates. On the basis of morphological and 16S rRNA sequence analysis, the isolated strain was identified as *L. plantarum*. In order to compare the obtained nucleotide sequence of strain MBP001 with the sequence database in GenBank, the BLASTn algorithm was used. A sequence identity of over 99% was observed between *L. plantarum* and nucleotide sequences in the database. Upon positive identification, the nucleotide sequence was deposited in NCBI’s GenBank database with accession number MZ496825.

A variety of qualitative and quantitative assays were conducted to confirm the production of a biosurfactant from isolated *L. plantarum* in MRS-Lac medium. In the first step, a drop collapse assay was performed, according to which surfactants destabilize droplets. A crude biosurfactant solution of *L. plantarum* was dispensed on oil in this manner. In the absence of a surfactant, the drops remain stable because the water molecules are repelled by hydrophobic sites. The drop will collapse if there is a surfactant present in the liquid, and the reason being a force or interfacial tension between the liquid and the hydrophobic surface. Flattened drops of a supernatant over oil showed the presence of a biosurfactant in the case of *L. plantarum*. Further, a confirmatory oil spreading assay was also performed to verify the results of the drop collapse assay. A direct relationship exists between oil displacement area and surfactant concentration in this assay. The oil spread test was performed using *L. plantarum* in relation to diameter and time, where a crude biosurfactant solution yielded positive results (Table 1).

Emulsification assays are used to screen the biosurfactant production in a quantitative manner. Biosurfactants are believed to emulsify the hydrocarbon substrates in mixture if they are present [36]. In this study, the emulsification of a crude *L. plantarum* biosurfactant was investigated against n-hexadecane. For *L. plantarum*, the crude biosurfactant solution demonstrated a 64.38 ± 1.47% emulsification index against n-hexadecane.

An alternative semiquantitative method to confirm biosurfactant production is the BAP assay. Dark blue halos that formed around the wells on BAP plates indicated the presence of a biosurfactant when a crude *L. plantarum* biosurfactant solution was added to the wells.

### 3.2. Antibacterial Activity

Since biosurfactants are known to possess antibacterial properties against a number of pathogenic bacteria [22,23], efforts were made to test the antibacterial potency of a crude *L. plantarum* biosurfactant against *P. aeruginosa* and *C. violaceum*. The antibacterial activity of the crude *L. plantarum* biosurfactant was assessed by measuring the MIC by broth microdilution assay. The MIC values of the crude *L. plantarum* biosurfactant against *P. aeruginosa* and *C. violaceum* were 6.25 and 3.12 mg/mL, respectively.

### 3.3. Effect of Crude L. plantarum Biosurfactant on QS-Regulated Virulence Factors

**Figure 1:**
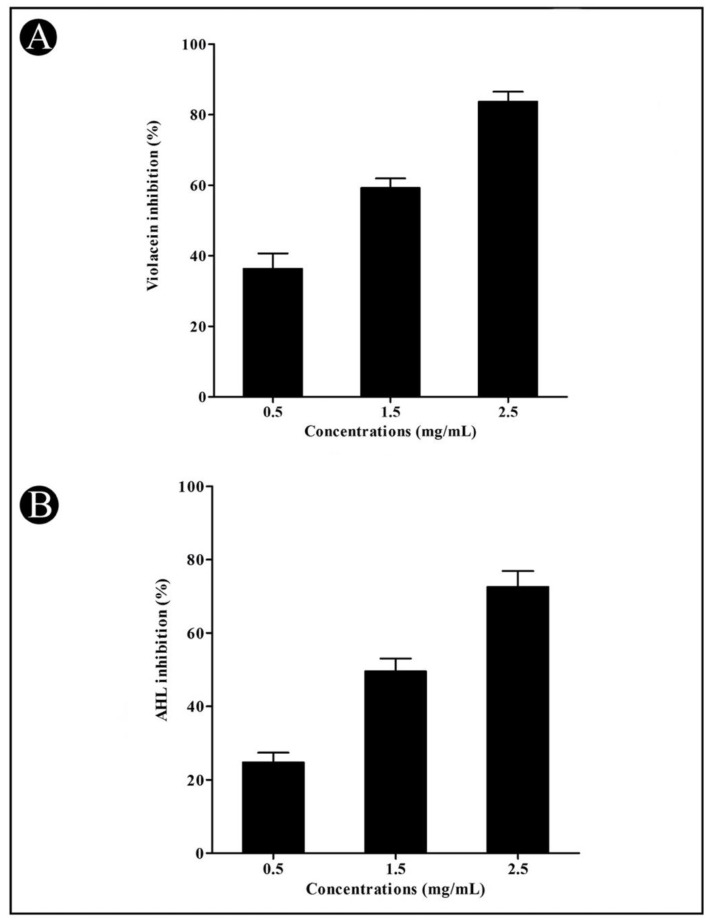
Inhibition of violacein and AHLs by the crude *L. plantarum* biosurfactant in *C. violaceum* at sub-MIC concentrations (0.5, 1.5, and 2.5 mg/mL). Violacein (**A**) and AHL (**B**) were detected by a UV–VIS spectrophotometer and presented as percentage inhibition (with respect to untreated control). Values are represented as mean ± SD of three independent experiments.

Initially, the crude *L. plantarum* biosurfactant was assessed against *C. violaceum* for its QS modulatory activity. A promising inhibition in pigment production was found in a dose-dependent approach at the sub-MIC level upon the treatment of the crude *L. plantarum* biosurfactant, which is an indicator of the inhibition of *C. violaceum* growth and anti-QS activity. By interfering with the QS activity, the crude biosurfactant reduced the production of *C. violaceum* pigment violacein. At sub-MIC concentrations of 0.5, 1.5, and 2.5 mg/mL, the crude biosurfactant inhibited violacein production by up to 36.29, 59.25, and 83.70%, respectively (Figure 1A). At the assessed sub-MIC concentrations, the crude biosurfactant did not show any inhibition in the growth of *C. violaceum*; instead, the production of a violacein pigment was inhibited significantly. The crude biosurfactant was also found to inhibit the AHL production in a dose-dependent approach at 24.77%, 49.55%, and 72.56% when compared with the control in the presence of 0.5, 1.5, and 2.5 mg/mL concentrations, respectively (Figure 1B). Overall, we found that the crude biosurfactant of *L. plantarum* significantly inhibited the AHL production in comparison with the control.

**Figure 2:**
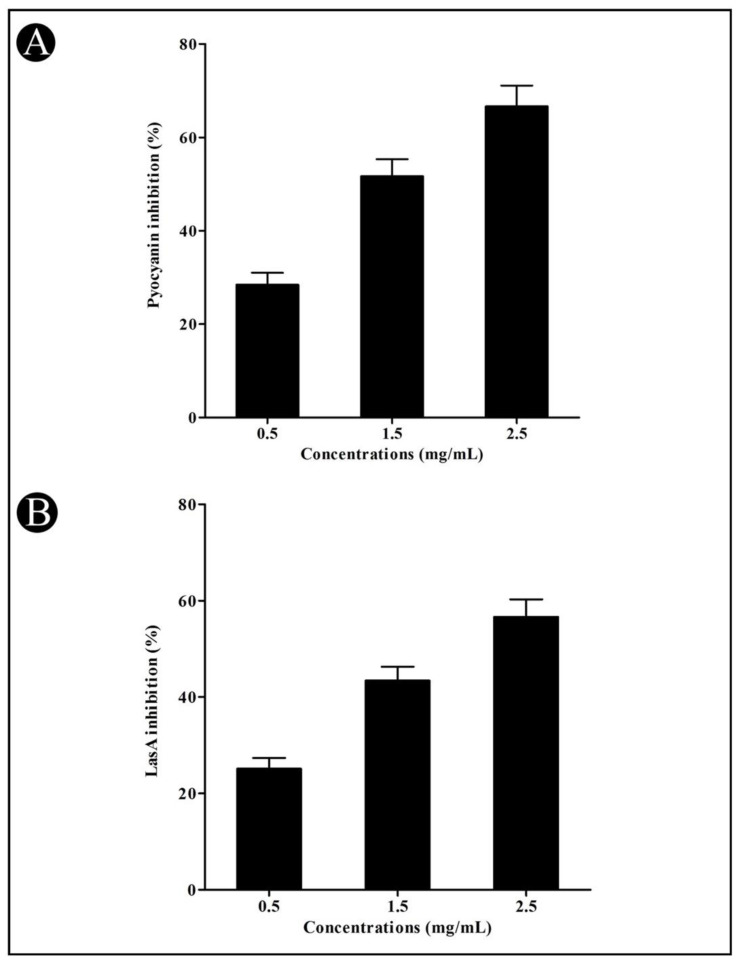
Inhibition of pyocyanin and LasA by the crude *L. plantarum* biosurfactant in *P. aeruginosa* at sub-MIC concentrations (0.5, 1.5, and 2.5 mg/mL). Pyocyanin (**A**) and LasA (**B**) were detected by a UV–VIS spectrophotometer and presented as percentage inhibition (with respect to untreated control). Values are represented as mean ± SD of three independent experiments.

In addition, the crude biosurfactant of *L. plantarum* was also studied for its anti-QS activity against *P. aeruginosa* by analyzing the virulent factors, such as LasA protease and LasB elastase activity and pyocyanin and azocasein degrading protease activity. Pyocyanin is produced by *P. aeruginosa*, which is one of the potent virulent factors associated with the virulence and pathogenesis of *P. aeruginosa*. It was noted that at sub-MIC concentrations, the crude *L. plantarum* biosurfactant was found to reduce the pyocyanin production in a dose-dependent manner (28.43%, 51.67%, and 66.63%) (Figure 2A). Having obtained promising results in the pyocyanin assay, we tested the inhibitory effects of the crude *L. plantarum* biosurfactant on the LasA protease activity of *P. aeruginosa*. As a result, in the presence of the crude biosurfactant at sub-MIC concentrations, we observed a concentration-dependent reduction in LasA protease activity (25.13, 43.37, and 56.62%, respectively) (Figure 2B).

**Figure 3:**
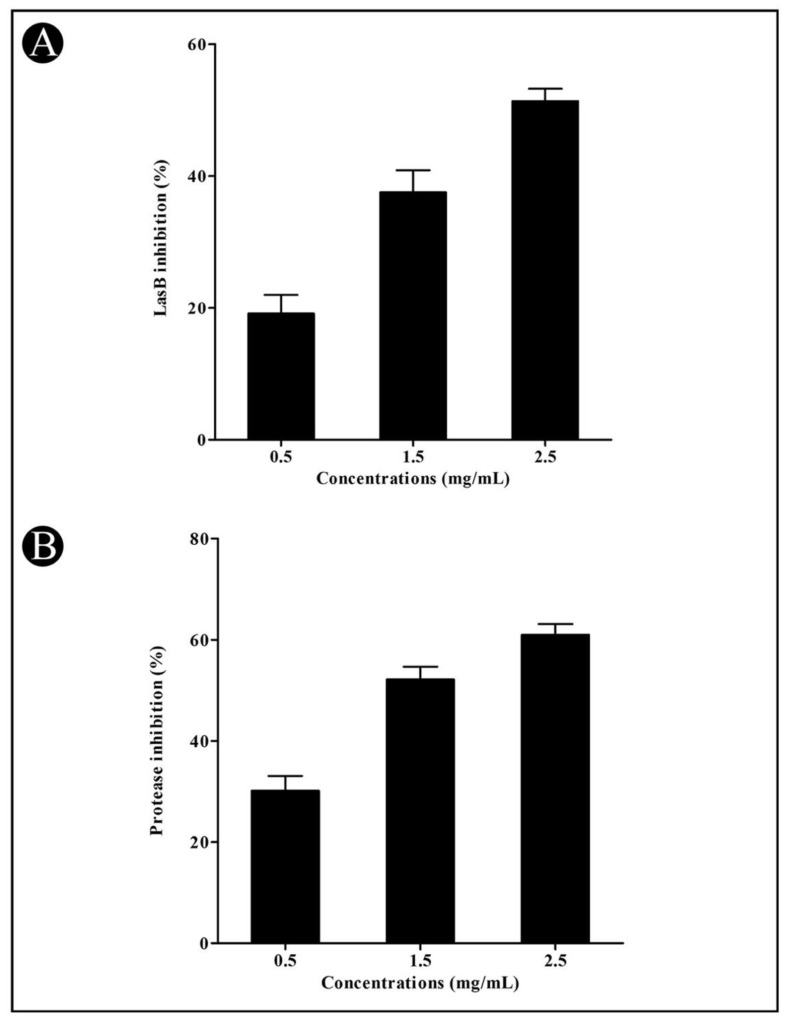
Inhibition of LasB and protease by the crude *L. plantarum* biosurfactant in *P. aeruginosa* at sub-MIC concentrations (0.5, 1.5, and 2.5 mg/mL). LasB (**A**) and activity of protease (**B**) were detected by a UV–VIS spectrophotometer and presented as percentage inhibition (with respect to untreated control). Values are represented as mean ± SD of three independent experiments.

Furthermore, the ability of LasB elastase to result in corneal ulcers, necrotic skin lesions, and pulmonary hemorrhages makes it a special and interesting enzyme to study. A significant reduction in LasB elastase activity was noted in a *P. aeruginosa* culture supernatant after treatment with the crude *L. plantarum* biosurfactant at sub-MIC concentrations in a concentration-dependent manner (19.13%, 37.51%, and 51.33%, respectively) (Figure 3A). The bacterial proteases are hydrolytic enzymes that cleave the proteins of host cells (infected skin), allowing the bacteria to invade and grow. In the present study, a crude biosurfactant of *L. plantarum* at sub-MIC concentrations was found effective in suppressing the bacterial proteinase production in a dose-dependent manner (30.13%, 52.18%, and 60.95%, respectively) (Figure 3B).

**Figure 4:**
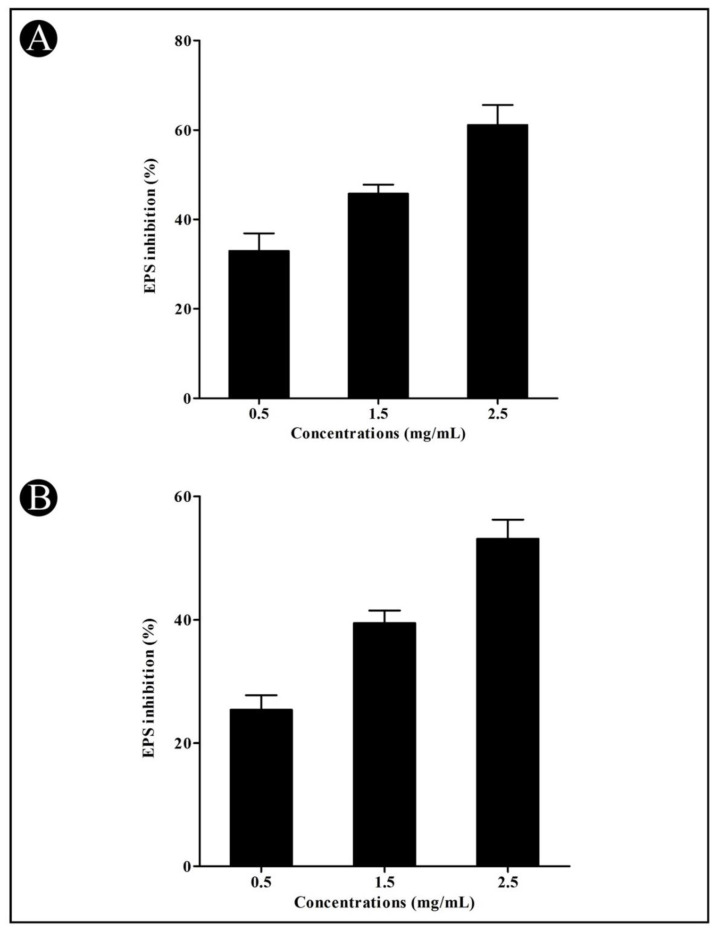
Inhibition of swarming motility and EPS production by the crude *L. plantarum* biosurfactant at a sub-MIC concentration. Effect of the crude biosurfactant on EPS production in *C. violaceum* (**A**) and *P. aeruginosa* (**B**) at sub-MIC concentrations (0.5, 1.5, and 2.5 mg/mL).

The rapid translocation of bacteria by swarming is conducive to promoting efficient colonization on surfaces. In comparison with untreated controls, crude *L. plantarum* biosurfactant-treated cells showed very little swarming motility in both *P. aeruginosa* and *C. violaceum*. EPS are bacterial biopolymers that are embedded in biofilms. In the biofilm, biopolymers of EPS form a matrix and retain water, keeping the cells together by retaining moisture. In the present study, a decrease in the EPS production (concentration dependent) was found in the treated culture of *C. violaceum* (32.95%, 45.74%, and 61.15%) and *P. aeruginosa* (25.40%, 39.45%, and 53.11%) and at sub-MIC concentrations (Figure 4A,B).

### 3.4. Antibiofilm Potential of Crude L. plantarum Biosurfactant

**Figure 5:**
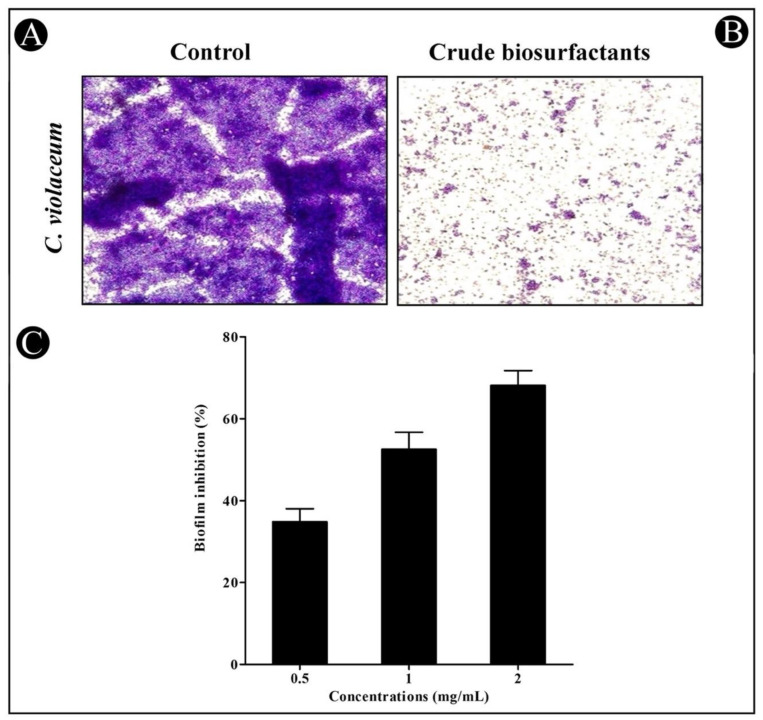
In situ light microscopy analysis of the biofilm inhibition and antibiofilm potential of the crude *L. plantarum* biosurfactant against *C. violaceum*. (**A**) The control shows biofilm formation. (**B**) The crude biosurfactant reduced the biofilm matrix at a concentration of 2.5 mg/mL. (**C**) The inhibition of the biofilm was detected by a UV–VIS spectrophotometer and presented as percentage inhibition (with respect to untreated control). Values are represented as mean ± SD of three independent experiments.

**Figure 6:**
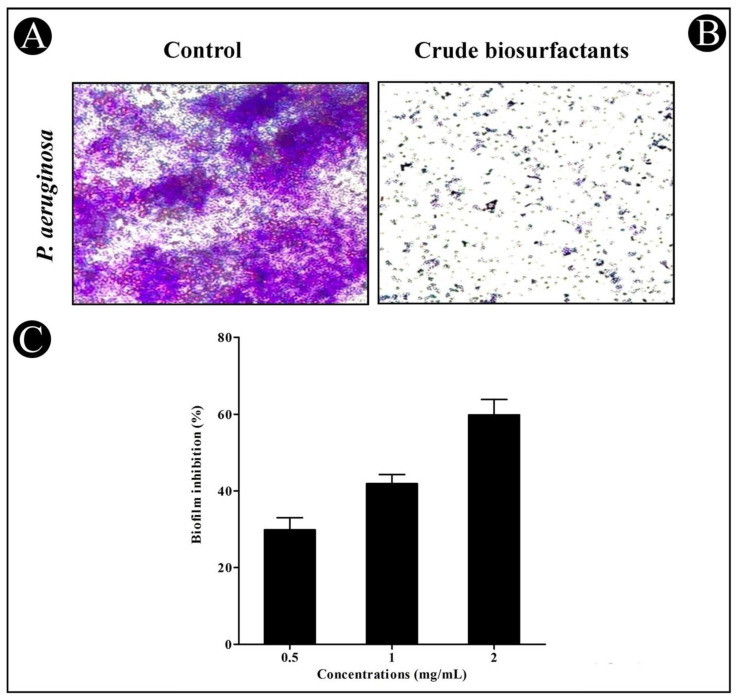
In situ light microscopy analysis of the biofilm inhibition and antibiofilm potential of the crude *L. plantarum* biosurfactant against *P. aeruginosa*. (**A**) The control shows biofilm formation. (**B**) The crude biosurfactant reduced the biofilm matrix at a concentration of 2.5 mg/mL. (**C**) The inhibition of the biofilm was detected by a UV–VIS spectrophotometer and presented as percentage inhibition (with respect to untreated control). Values are represented as mean ± SD of three independent experiments.

The crude *L. plantarum* biosurfactant’s antibiofilm potential at sub-MIC concentrations was determined by crystal violet assay. Our results presented that the crude *L. plantarum* biosurfactant efficiently inhibited the formation of biofilms in a dose-dependent approach in both test strains (Figure 5 and Figure 6). At sub-MIC concentrations, biofilm inhibition in *C. violaceum* was found to be 34.87%, 52.53%, and 68.12% (Figure 5C), while in *P. aeruginosa*, it was found to be 29.86%, 41.87%, and 59.80% (Figure 6C). Furthermore, the efficacy of the crude *L. plantarum* biosurfactant in disrupting the biofilms of test strains was also examined using LM. Under LM, deterioration in the thickness of biofilms with lower microcolonies’ appearance was observed in the presence of the crude *L. plantarum* biosurfactant (Figure 5B and Figure 6B), when compared with the control (Figure 5A and Figure 6A), where a heavy-knit-like mat of biofilms was observed.

### 3.5. Identification of Chemical Constituents by GC–MS

The chemical constituents in the crude biosurfactant of *L. plantarum* were characterized via GC–MS. Consequently, the identified and characterized chemical constituents are represented in Table 2. The total ion chromatogram of the crude biosurfactant of *L. plantarum* confirmed the presence of different biologically active compounds with different retention times (Figure S1–Supplementary). Structural complexities of the compounds were identified with the help of mass spectrometry (MS) analysis. The identified chemical constituents in the crude biosurfactant of *L. plantarum* were octane, 2,4,6-trimethyl; undecane; dodecane; hexadecane; n-hexadecanoic acid; oleic acid; ethyl oleate; octadecanoic acid, ethyl ester; 3,7,11,15-tetramethylhexadec-2-en-1-yl acetate; 1-hexadecyl-2,3-dihydro; hexadecanoic acid; 1H-indene, 2-hydroxy-1-hydroxymethyl; and octadecanoic acid, 2,3-dihydroxypropyl ester.

### 3.6. Docking Analysis

**Figure 7:**
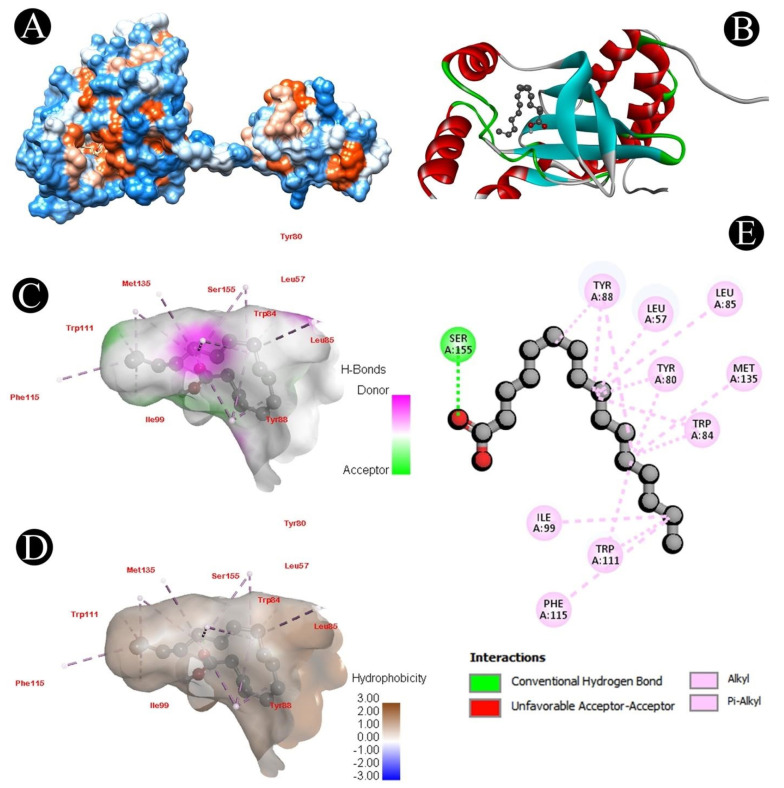
Visualization of the docking analysis of n-hexadecanoic acid binding with 3QP5. (**A**) Interaction of n-hexadecanoic acid with 3QP5. (**B**) Close-up of the interaction of n-hexadecanoic acid with 3QP5. (**C**) Visualization of hydrogen bond. (**D**) Visualization of hydrophobic interaction. (**E**) Two-dimensional representation describing the bindings of n-hexadecanoic acid with an active site of 3QP5.

**Figure 8:**
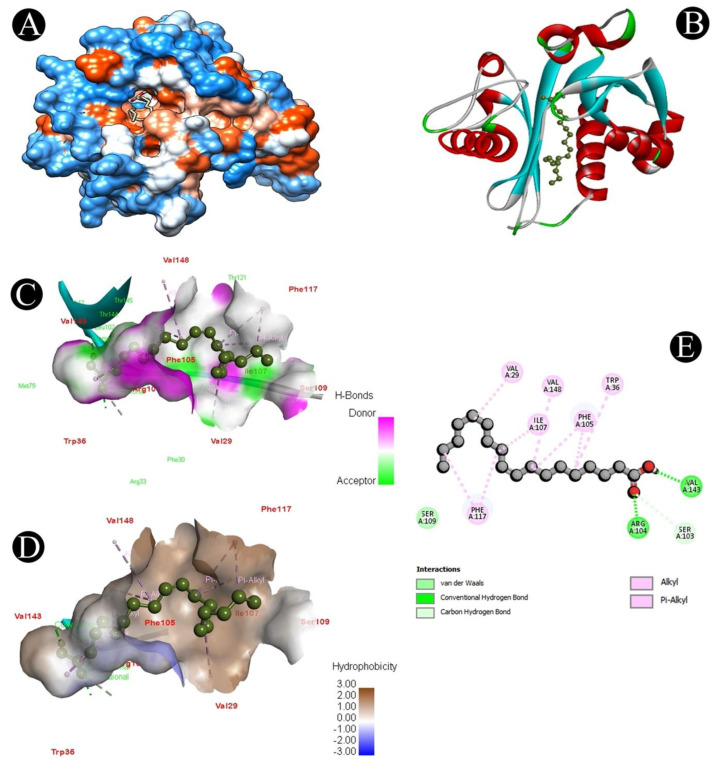
Visualization of the docking analysis of oleic acid binding with 1RO5. (**A**) Hydrophobicity surface 3D representation. (**B**) Interaction of oleic acid with 1RO5. (**C**) Visualization of hydrogen bond. (**D**) Visualization of hydrophobic interaction. (**E**) Two-dimensional representation describing the bindings of oleic acid with an active site of 1RO5.

**Figure 9:**
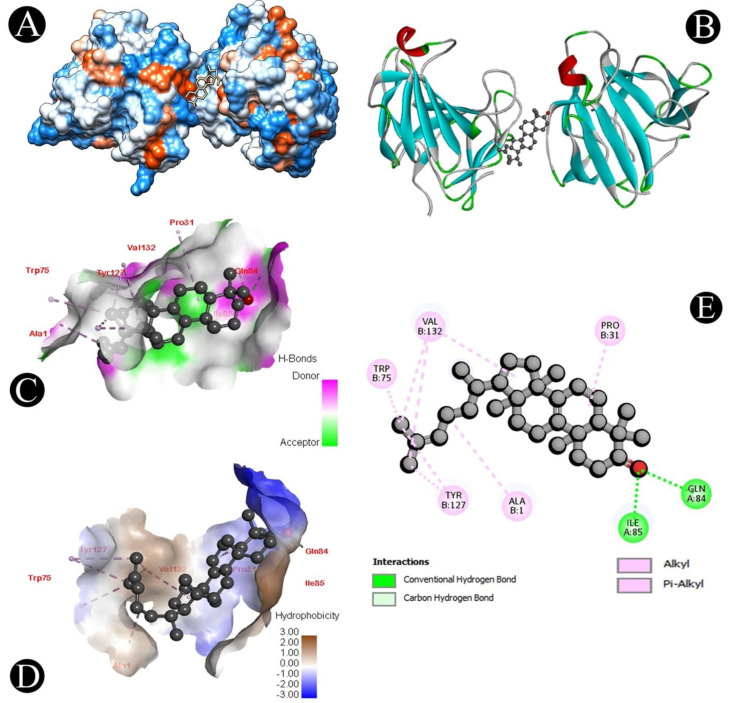
Visualization of the docking analysis of 1H-indene,1-hexadecyl-2,3-dihydro binding with 3IT7. (**A**) Hydrophobicity surface 3D representation. (**B**) Interaction of 1H-indene,1-hexadecyl-2,3-dihydro with 3IT7. (**C**) Visualization of hydrogen bond. (**D**) Visualization of hydrophobic interaction. (**E**) Two-dimensional representation describing the bindings of 1H-indene,1-hexadecyl-2,3-dihydro with an active site of 3IT7.

**Figure 10:**
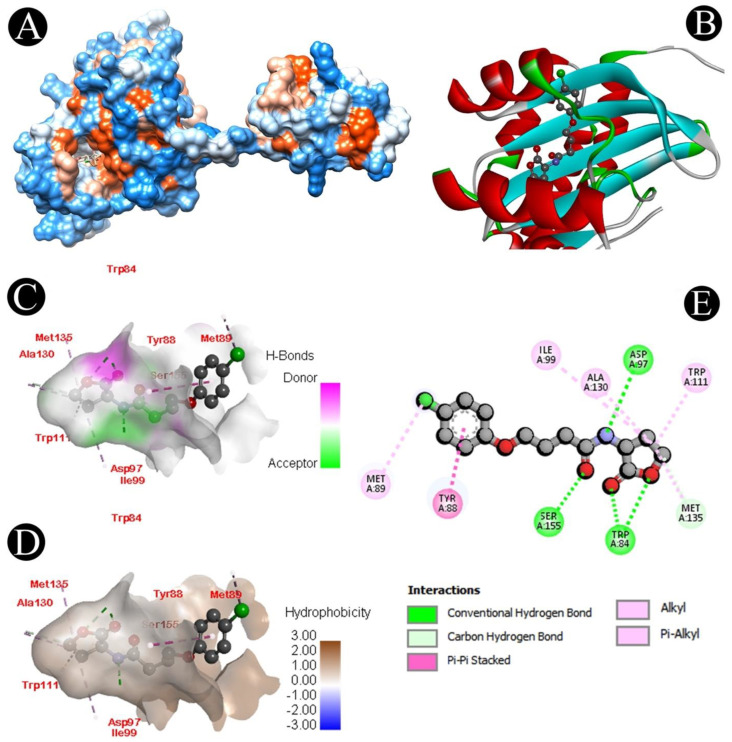
Visualization of the docking analysis of the standard QS inhibitor chlorolactone binding with 3QP5. (**A**) Interaction of chlorolactone with 3QP5. (**B**) Close-up of the interaction of chlorolactone with 3QP5. (**C**) Visualization of hydrogen bond. (**D**) Visualization of hydrophobic interaction. (**E**) Two-dimensional representation describing the bindings of chlorolactone with an active site of 3QP5.

The LasI and CviR genes play an important role in QS, biofilm formation, and the survival of *P. aeruginosa* and *C. violaceum*, respectively. Different chemical compounds identified from the crude biosurfactant of *L. plantarum* via GC–MS analysis were subjected to molecular docking studies to predict the binding score (Table 3). From all the identified compounds, n-hexadecanoic acid had a high affinity for the CviR receptor protein with a binding energy of −7.9 kcal/mol, while oleic acid had a high affinity for the LasI receptor protein with a binding energy of −7.3 kcal/mol, and 1H-indene,1-hexadecyl-2,3-dihydro had a high affinity for the LasA receptor protein with a binding energy of −7.5 kcal/mol. The active site covered by both compounds in different ways can be observed in Figure 7, Figure 8, Figure 9 and Figure 10. Binding energies of all the identified compounds in the biosurfactant with the target proteins are in line with the binding energy of the standard QS inhibitor.

## 4. Discussion

A biofilm-related infection is often difficult to treat due to the resistance of bacteria to various antibiotics, and approximately 65% of all bacterial infections have been associated with bacterial biofilms [37]. Therefore, it is important to identify new and effective molecules that can prevent the formation of biofilms [4]. Moreover, antibiotic therapy is insufficient to fight biofilm-related infections; however, understanding the mechanism of biofilm formation will help in strategically controlling and combatting the biofilm infections [6]. In addition, inhibiting biofilms is one of the first lines of defense that regulates the growth and survival of surface-adhered bacteria. In many bacteria, certain behaviors, such as, the formation of a biofilm and virulence potential, are modulated through a cascade of signaling happenings known as QS [38]. Obstruction with QS cascades is believed to be a potential approach to reduce bacterial virulence and pathogenicity. It is believed that when bacterial species reach a certain density after communicating through signal molecules, they initiate the expression of virulence genes, which enables the synthesis of virulence factors. Therefore, blocking the communication between bacteria constitutes one of the new targets that can be achieved in antimicrobial therapy [39].

Compounds with QSI effect are the new-generation antimicrobial agents [40]. Recent research studies have focused on the use of biosurfactants due to their excellent selective and specific mechanism of action under harsh environmental conditions, such as temperature, pH, and salinity, as well as environmentally friendly nature [41]. There are many applications for biosurfactants in a variety of fields, including food, lubricants, biodegradation, cosmetics, agriculture, pesticides, and medicine/pharmaceuticals [42,43]. Furthermore, biosurfactants have also shown to be effective in the fight against pathogenic bacteria and, therefore, their biofilms [44]. The antimicrobial activity of various strains of LAB and their ability to inhibit the formation of the biofilms of many pathogenic microbes have been reported [45,46,47,48,49,50,51,52,53,54]. Probiotics open a new horizon in the fight against biofilms. Since probiotics are less cytotoxic than anti-QS agents and have a natural effect, they can be considered the ideal choice in combating biofilms formed by pathogenic bacterial species [6]. As a result, to search for a novel method/molecule that can be used for the prevention of biofilm formation, we investigated the anti-QS and antibiofilm activity of the crude biosurfactant produced by *L. plantarum* against the model pathogenic organisms *C. violaceum* and *P. aeruginosa*, as it has been reported that biosurfactants exhibit antimicrobial activity [22,23].

*C. violaceum* is a suitable indicator organism for the screening of QS inhibitors because of its purple pigment (violacein), which is easily detectable. In this study, a crude biosurfactant of *L. plantarum* was evaluated for QS interference in *C. violaceum* by assessing the reduction in violacein pigment production. Violacein production is controlled by a CviR-dependent QS system in *C. violaceum* [48]. As a consequence, any alteration in *C. violaceum* pigment production is interpreted as a direct clue of QS hindrance. Similar to the crude *L. plantarum* biosurfactant, other natural products are also reported to inhibit violacein production in *C. violaceum* [55,56]. Furthermore, in the present study, a crude biosurfactant of *L. plantarum* showed the inhibition of AHL molecules, which indicated that the crude biosurfactant interfered with QS by inhibiting the synthesis of AHLs. Accordingly, based on the violacein inhibition study, we found the crude biosurfactant of *L. plantarum* to be the most active, and thus, we proceeded with the further screening of the crude biosurfactant against *P. aeruginosa* to evaluate the broad-spectrum QS activity, since the *P. aeruginosa* QS system is entirely different from *C. violaceum*.

We determined the effect of the crude biosurfactant of *L. plantarum* on various QS-mediated phenotypes of *P. aeruginosa*, such as azocaseinolytic activity, synthesis of virulent factors such as pyocyanin, LasA protease, and LasB elastase activity [30]. Virulence factors are known to play an important role in the invasion of bacteria and their proliferation in the host population [30]. It has been documented that *P. aeruginosa* secretes proteases in order to cause the progression of pathogenesis [30]. Obtained results of the azocasein degrading assay indicated that the crude biosurfactant of *L. plantarum* at sub-MIC concentrations reduced the protease production of *P. aeruginosa*. In *P. aeruginosa*, two QS systems (LasIR and RhIIR) are found [57]. In the first QS system (las), the diffusible signal molecules 3O-C12-HSL are formed due to *lasI*-encoded synthase, which further interacts with LasR (transcriptional activator) to activate many virulence genes (*lasB* and *lasA*). These genes are involved in the production of various virulent enzymes, such as alkaline protease, LasA protease, and LasB elastase [58,59].

In the second QS system (rhl) of *P. aeruginosa*, the diffusible signal molecules C4-HSL are synthesized due to the rhlI, which is further associated with RhlR and activates the expression system for pyocyanin production. In the pathogenesis of *P. aeruginosa*, LasIR-encoded proteins play a crucial role in protease and elastase production [29]. In this study, we assessed different sub-MIC concentrations of the crude biosurfactant of *L. plantarum*, which inhibited all the QS-associated proteins and factors in a dose-dependent approach. Another important and different virulence factor, which is produced under QS regulation, is pyocyanin. A role for pyocyanin in pathogenesis is also well documented, especially in cystic fibrosis [60]. Our results are in agreement with those in the literature, where other natural products were reported to inhibit QS-associated proteins and factors of *P. aeruginosa* to varying degrees (10–90%) [61].

In one study, it was found that the production of lactic acid, short-chain N-Acyl homoserine lactones (AHL) produced by probiotics, has an inhibitory effect on QS by suppressing the biofilm formation of *P. aeruginosa* [62]. Again, *L. helveticus*, *L. lactis*, and *L. casei* strains showed similar inhibitory effects on *E. coli* O157:H7, *Salmonella typhimurium*, and *L. monocytogenes* pathogens [63]. Organic acids synthesized by probiotics can also act as QS antagonists, which can inhibit AHL production at the gene expression level and stop the formation of biofilms [6]. Similarly, *L. brevis* with strong probiotic properties was also found to regulate the QS system [64]. Onbas et al. (2019) showed the antimicrobial, antibiofilm, anti-QS, and antioxidant activity by the probiotic *L. plantarum* F-10 strain [39]. Similar to our study, the antibiofilm effect of biosurfactants isolated from *L. casei* on *S. aureus* strains was also studied [65]. It is further reported that *L. reuteri*, *L. casei*, *L. salivarius*, and *L. plantarum* strains can inhibit the biofilm formation and expression of QS-related genes in *Streptococcus mutans* [66].

Other important factors connected to the development of biofilms in bacteria, such as swarming motility and production of EPS, were also assessed in the present study. The production of EPS is a QS-dependent trait that is necessary for the maturation of biofilms [67]. As a result, EPS production is reduced because a crude biosurfactant interferes with QS. Therefore, it is hoped that a crude biosurfactant that dramatically reduces EPS may be able to reduce the resistance level of the pathogen in sessile mode. In addition, there was a noticeable decrease in the swarming motility of the tested bacteria. In biofilm development, flagellar-driven motility has been shown to be necessary for the initiation of surface attachment [68]. Consequently, inhibiting flagellar synthesis with crude biosurfactants would reduce the swarming migration. This suggests that crude biosurfactants may have indirectly affected the biofilm formation of bacteria by disrupting the AHL-mediated QS system.

It is very well known that the architecture of biofilms of Gram-negative bacterial pathogens associated with human infections is regulated and coordinated by the QS system of bacteria. QS also plays an essential role in the drug resistance and pathogenesis of the disease, besides its key involvement in the attachment, establishment, and maturation of sessile biofilms. Therefore, interference with the QS system may lead to interference with the establishment of the biofilm matrix and the subsequent dissemination of the infection within the host cells. Using crystal violet staining to stain the biofilm cells, we were able to confirm the inhibitory effect of the *L. plantarum*-derived crude biosurfactant on the *C. violaceum* and *P. aeruginosa* biofilms. In order to confirm this, we further used LM to examine the biofilms with disintegrated architectures at sub-MIC concentrations. The results obtained in our study are in agreement with the activity of other natural products, such as *Lagerstroemia speciosa* fruit extract, *Sclerocarya birrea* bark extract, *Conocarpus erectus*, *Chamaesyce hypericifolia*, *Callistemon viminalis*, *Bucida buceras*, *Tetrazygia bicolor*, and *Quercus virginiana* plant extracts [30,69,70].

We further validated the QS-mediated biofilm inhibition with the crude extract of *L. plantarum* by studying the molecular interactions of QS-associated CviR, LasI, and LasA proteins with the identified bioactive compounds via GC–MS analysis. Proteins involved in QS were docked with the identified compounds, which displayed that n-hexadecanoic acid binds to the ligand pocket of CviR, oleic acid binds to the ligand pocket of LasI, and 1H-indene,1-hexadecyl-2,3-dihydro binds to the ligand pocket of Las A with a high binding affinity. Recently, several studies reported that fatty acids exhibit antihyphal, antibiofilm, and anti-QS activities at concentrations less than their MICs [71]. For example, several fatty acids have been shown to selectively disrupt or inhibit biofilms of various microbial pathogens, such as *C. violaceum*, *S. aureus* [72,73], *P. aeruginosa* [74], and *C. albicans* [75,76]. Some monounsaturated fatty acids, such as palmitoleic (*cis*-9-hexadecenoïc) and myristoleic (*cis*-9-tetradecenoïc) acids, were shown to inhibit several genes’ expression in *V. cholerae* [77,78]. These molecules also prevent the interaction between their transcriptional regulator and the DNA [79]. Besides, monounsaturated fatty acids can also affect virulence factor expression, initial adhesion, or motility [80]. The anti-QS and antibiofilm efficacy of the unsaturated fatty acids palmitoleic (*cis*-9-hexadecenoïc) and myristoleic (*cis*-9-tetradecenoïc) has been reported against *A. baumannii* [81]. This implies that a crude *L. plantarum* biosurfactant may target the QS-related proteins for antibiofilm activity.

## 5. Conclusions

Collectively, this study revealed that a crude *L. plantarum* biosurfactant has significant anti-QS and antibiofilm potential against Gram-negative pathogenic bacteria. The active compounds in the identified chemical constituents of a crude *L. plantarum* biosurfactant can individually be further explored as an alternative to synthetic antibiotics, toxic antibiofilm, and anti-QS agents. A biosurfactant extracted from *L. plantarum* can possibly hamper the formation of biofilms and obstruct with the AHL-based QS-controlled virulence factors in *P. aeruginosa* and *C. violaceum.* Biological-source-derived biosurfactants are promising, and their favorable properties facilitate their use as an efficient tool in various applications. In order to provide a complete picture and perspectives for potential growth and practical applications, understanding of biosurfactant-producing strains needs to be extended, and the influence of various physicochemical and biochemical parameters on biosurfactants must be studied, which will also help in minimizing the production costs. More attention should also be aimed at extremophilic and hyperextremophilic biosurfactant-producing microorganisms to allow the use in numerous conditions. Further investigations on crude biosurfactants will provide a better understanding of the therapeutic potential and broad environmental and pharmacological usages before efforts are made to develop their real-time use in various applications, especially for pharmaceutical development.

## Figures and Tables

**Table 1:** Qualitative and quantitative results of different screening assays for the production of a biosurfactant (values are representatives of mean ± SD (n = 3)).

Strain	Colony Characteristics	Gram’s Reaction	Oil Spreading Test	Drop Collapse Test	BAP Test	%E24 (n-Hexadecane)
***L. plantarum*-MBP001**	Creamy, small, circular, slightly raised, entire, smooth	Gram-positive, rod shaped	Positive	Positive	Positive	64.38 ± 1.47

**Table 2:** Major constituents in the crude biosurfactant of *L. plantarum* using GC–MS.

No.	RT	% Area	Compound Name	Class	Structure
1	5.784	0.30	Octane, 2,4,6-trimethyl-	Fatty acyl	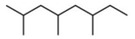
2	6.686	0.32	Undecane	Fatty acyl	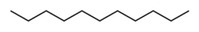
3	7.500	0.90	Dodecane	Fatty acyl	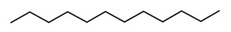
4	8.936	0.27	Hexadecane	Fatty acyl	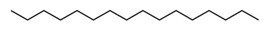
5	12.146	7.29	n-Hexadecanoic acid	Fatty acid	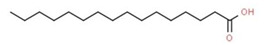
6	13.015	6.88	Oleic Acid	Fatty acid	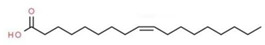
7	13.149	3.30	Ethyl Oleate	Fatty acid	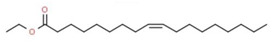
8	13.256	0.40	Octadecanoic acid, ethyl ester	Fatty acid	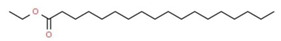
9	13.350	0.30	3,7,11,15-Tetramethylhexadec-2-en-1-yl acetate	Prenol lipid	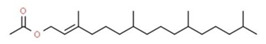
10	14.256	1.55	1H-indene, 1-hexadecyl-2,3-dihydro-	Fatty acyl	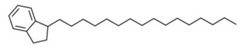
11	14.848	2.23	Hexadecanoic acid, 2-hydroxy-1-(hydroxymethyl) ethyl ester	Glycerolipid	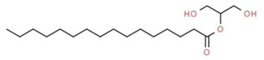
12	16.297	0.74	Octadecanoic acid, 2,3-dihydroxypropyl ester	Glycerolipid	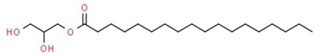

**Table 3:** Binding affinities of the top-rated pose of the ligand–receptor complex.

Compound Name	3QP5	1RO5	3IT7
Chlorolactone (standard QS inhibitor)	−8.1	−7.6	−7.8
Octane, 2,4,6-trimethyl-	−6.1	−6.3	−5.6
Undecane	−5.5	−5.6	−5.0
Dodecane	−5.6	−5.7	−4.8
Hexadecane	−6.0	−5.2	−5.0
n-Hexadecanoic acid	−7.9	−5.1	−4.6
Oleic Acid	−6.6	−7.3	−5.1
3,7,11,15-Tetramethylhexadec-2-en-1-yl acetate	−6.7	−5.9	−5.9
1H-indene, 1-hexadecyl-2,3-dihydro-	−7.6	−6.4	−7.5

## Data Availability

All data generated or analyzed during this study are included in this article.

## Supplementary Materials

The following supporting information can be downloaded at: https://www.mdpi.com/article/10.3390/microorganisms10051026/s1, Figure S1: GC-MS analysis of crude biosurfactant derived from *L. plantarum*.
