# Supplementary figures and images for: Lactiplantibacillus plantarum-Derived Biosurfactant Attenuates Quorum Sensing-Mediated Virulence and Biofilm Formation in Pseudomonas aeruginosa and Chromobacterium violaceum
Source: Microorganisms. 2022 May 13;10(5):1026. doi: 10.3390/microorganisms10051026 (PMC9145448; doi:10.3390/microorganisms10051026)

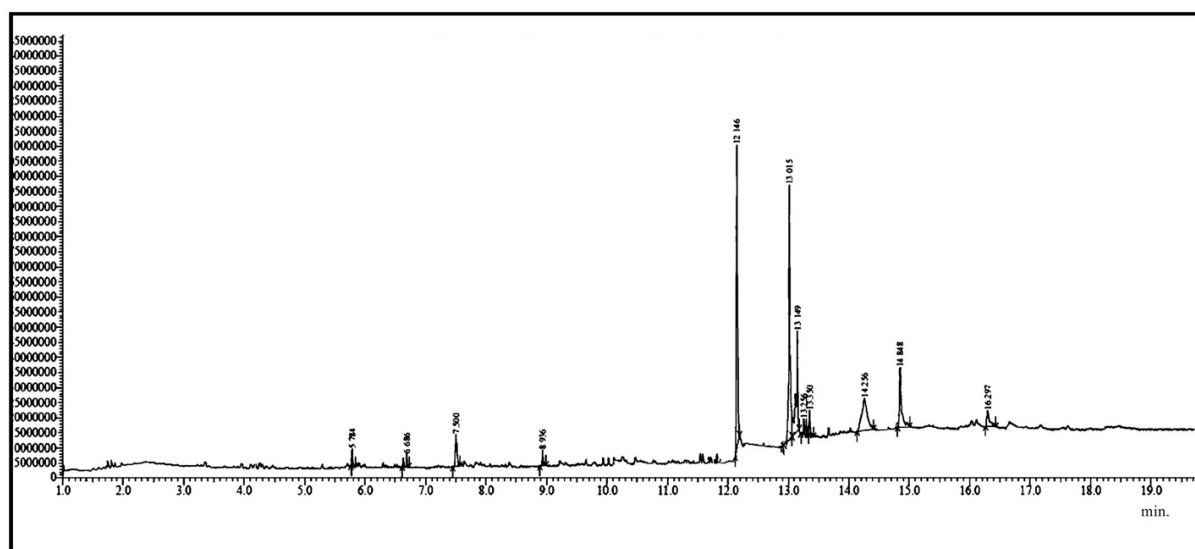

**Figure S1.** GC-MS analysis of crude biosurfactant derived from *L. plantarum*.

Supplement: Supplementary file 1 [file microorganisms-10-01026-s001.zip › microorganisms-1694085-supplementary.pdf]
